# Body size mediates latitudinal population differences in the response to chytrid fungus infection in two amphibians

**DOI:** 10.1007/s00442-023-05489-5

**Published:** 2023-12-14

**Authors:** Sara Meurling, Mattias Siljestam, Maria Cortazar-Chinarro, David Åhlen, Patrik Rödin-Mörch, Erik Ågren, Jacob Höglund, Anssi Laurila

**Affiliations:** 1 Animal Ecology/ Department of Ecology and Genetics, Uppsala University, Uppsala, Sweden; 2 MEMEG/Department of Biology, Lund University, Lund, Sweden; 3 Department of Earth, Ocean and Atmospheric Sciences, University of British Columbia, Vancouver, Canada; 4 Department of Ecology, Environment and Plant Sciences, Stockholm University, Uppsala, Sweden; 5 Department of Pathology and Wildlife Diseases, National Veterinary Institute, Uppsala, Sweden

**Keywords:** *Batrachochytrium dendrobatidis*, Body size, Geographical variation, Sublethal effects, Tolerance

## Abstract

Factors behind intraspecific variation in sensitivity to pathogens remain poorly understood. We investigated how geographical origin in two North European amphibians affects tolerance to infection by the chytrid fungus *Batrachochytrium dendrobatidis* (*Bd),* a generalist pathogen which has caused amphibian population declines worldwide. We exposed newly metamorphosed individuals of moor frog *Rana arvalis* and common toad *Bufo bufo* from two latitudinal regions to two different *Bd*GPL strains. We measured survival and growth as infections may cause sub-lethal effects in fitness components even in the absence of mortality. Infection loads were higher in *B. bufo* than in *R. arvalis,* and smaller individuals had generally higher infection loads. *B. bufo* had high mortality in response to *Bd* infection, whereas there was little mortality in *R. arvalis*. *Bd*-mediated mortality was size-dependent and high-latitude individuals were smaller leading to high mortality in the northern *B. bufo*. *Bd* exposure led to sub-lethal effects in terms of reduced growth suggesting that individuals surviving the infection may have reduced fitness mediated by smaller body size. In both host species, the Swedish *Bd* strain caused stronger sublethal effects than the British strain. We suggest that high-latitude populations can be more vulnerable to chytrids than those from lower latitudes and discuss the possible mechanisms how body size and host geographical origin contribute to the present results.

**Supplementary Information:**

The online version contains supplementary material available at 10.1007/s00442-023-05489-5.

## Introduction

Natural populations are increasingly affected by emerging infectious diseases (Daszak et al. 2000; Pennisi 2010; Fisher et al. 2012; Scheele et al. 2019). Many of the emerging diseases are caused by generalist and opportunistic fungal pathogens which can infect a wide range of host species (Wibbelt et al. 2010; Fisher et al. 2012; Lorch et al. 2016; More et al. 2018). The virulence of fungal pathogens often differs among host species, leading to population declines in some hosts while having no apparent effect on others (Casadevall 2007; Herczeg et al. 2021). There is evidence that populations of a host species may differ in their susceptibility, but apart from plant systems few studies have addressed this question in detail (Ebert 2008; Laine et al. 2011; Bradley et al. 2015; Martin-Torrijos et al. 2017).

The chytrid fungus *Batrachochytrium dendrobatidis* (*Bd*), causing the disease chytridiomycosis in amphibians, is a generalist pathogen which has caused the decline of over 500 amphibian species, including the presumed extinction of 90 species (Berger et al. 1998; Skerratt et al. 2007; Lips 2016; Scheele et al. 2019). *Bd* is endemic to East Asia, and severe outbreaks of chytridiomycosis have been observed in the Americas and Australia (Lips 2016; O’Hanlon et al. 2018; Scheele et al. 2019). There is considerable variation in virulence among genetic strains of *Bd* (Farrer et al. 2011; Bataille et al. 2013; Greenspan et al. 2018) and *Bd*GPL, the global panzootic lineage originating in Eastern Asia, has caused most of the chytridiomycosis outbreaks (O’Hanlon et al. 2018). While genetic variation within *Bd*GPL is relatively limited (O’Hanlon et al. 2018), there is evidence for virulence differences also between *Bd*GPL strains (Becker et al. 2017; Burrow et al. 2017; Dang et al 2017, Greener 2020).

As a generalist pathogen, *Bd* infects a wide range of amphibian species (Lips 2016; Scheele et al. 2019). Not all species develop chytridiomycosis; many are resistant to the disease and can clear the infection, while others can tolerate high infection loads without developing the disease (Fisher et al. 2009a; Gahl et al. 2012; Ellison et al. 2014; Scheele et al. 2017). Similarly, geographical populations of the same species can differ in their susceptibility to *Bd* (Savage and Zamudio 2011; Bradley et al. 2015; Kosch et al. 2019). These differences can be due to genetic differences in traits like immune response and behavior (Richards-Zawacki 2010), and are in some cases linked with direct *Bd*-mediated selection (Savage and Zamudio 2016; Savage et al. 2018). Although infection does not cause direct mortality in the resistant and tolerant populations and species, sub-lethal fitness effects such as decreased growth have been detected (Bielby et al. 2015; Burrow et al. 2017).

Climate-related latitudinal divergence is an important structuring force of intraspecific genetic variation (e.g., Hewitt 2000, Conover et al. 2009), but its potential role in mediating host–pathogen interactions has received little attention. Two lines of evidence suggest that amphibian populations living at high latitudes in the northern hemisphere may be especially vulnerable to disease. Firstly, due to post-glacial colonization patterns northern populations often harbor less genetic variation (Hewitt 2000). In many amphibians, this is true also for immunogenetic variation in major histocompatibility (MHC) genes (Zeisset and Beebee 2014; Cortázar-Chinarro et al. 2017, 2022; Höglund et al. 2022), which is associated with *Bd* resistance (Savage and Zamudio 2011; Savage et al. 2018; Kosch et al. 2019). Furthermore, pathogen species richness and abundance are significant predictors of adaptive MHC variation (Wang et al. 2017). As pathogen richness and abundance decrease towards colder climates (Schemske et al. 2009), populations at higher latitudes may encounter lower diversity and a lower number of pathogens which may lead to increased drift and loss of adaptive immunogenetic variation in these populations (Cortázar-Chinarro et al. 2017). Secondly, time-constrained high-latitude environments select for high larval development rates (Palo et al. 2003; Luquet et al. 2019), which in amphibians can trade-off with disease resistance (Johnson et al. 2011; Woodhams et al. 2016) and immune response (Gervasi and Foufopoulos 2007; Murillo-Rincon et al. 2017). While all these factors may contribute to lower ability to withstand novel pathogens in high-latitude populations, no studies on disease resistance between latitudinal populations have been made.

*Bd* is widely spread in southern Scandinavia (e.g., Meurling et al. 2020), but very few experimental studies focusing on sensitivity of high-latitude amphibians to *Bd* infection have been made (Cortazar-Chinarro et al. 2022). Here we conducted a laboratory common garden experiment to examine inter- and intraspecific population differences in response to *Bd* infection in Scandinavian amphibians. Our aims were three-fold. First, we investigated the responses of two common north European amphibians (moor frog *Rana arvalis* and common toad *Bufo bufo*) to *Bd* infection. Second, we investigated if the responses differ between southern and northern Scandinavian populations of these species. We predicted that due to lower genetic variation and higher development rates in the north, the northern populations are more sensitive to *Bd* infection. Finally, we evaluated if the amphibian responses to infection differ between two geographically separated *Bd* lineages. To this end, we infected newly metamorphosed amphibians and measured their survival and growth during a 30-day period.

## Methods

### Animal rearing

Both *R. arvalis* (hereafter *Ra*) and *B. bufo* (hereafter *Bb*) are widespread amphibians in Europe occurring up to the polar circle in the north (Sillero et al. 2014). Both species are explosive breeders and mate in early spring. In southern Sweden, *Bd* prevalence in breeding adults is 15.3% (n = 288) and 3.4% (n = 941) in *Ra* and* Bb*, respectively (Meurling et al. 2020).

**Fig. 1:**
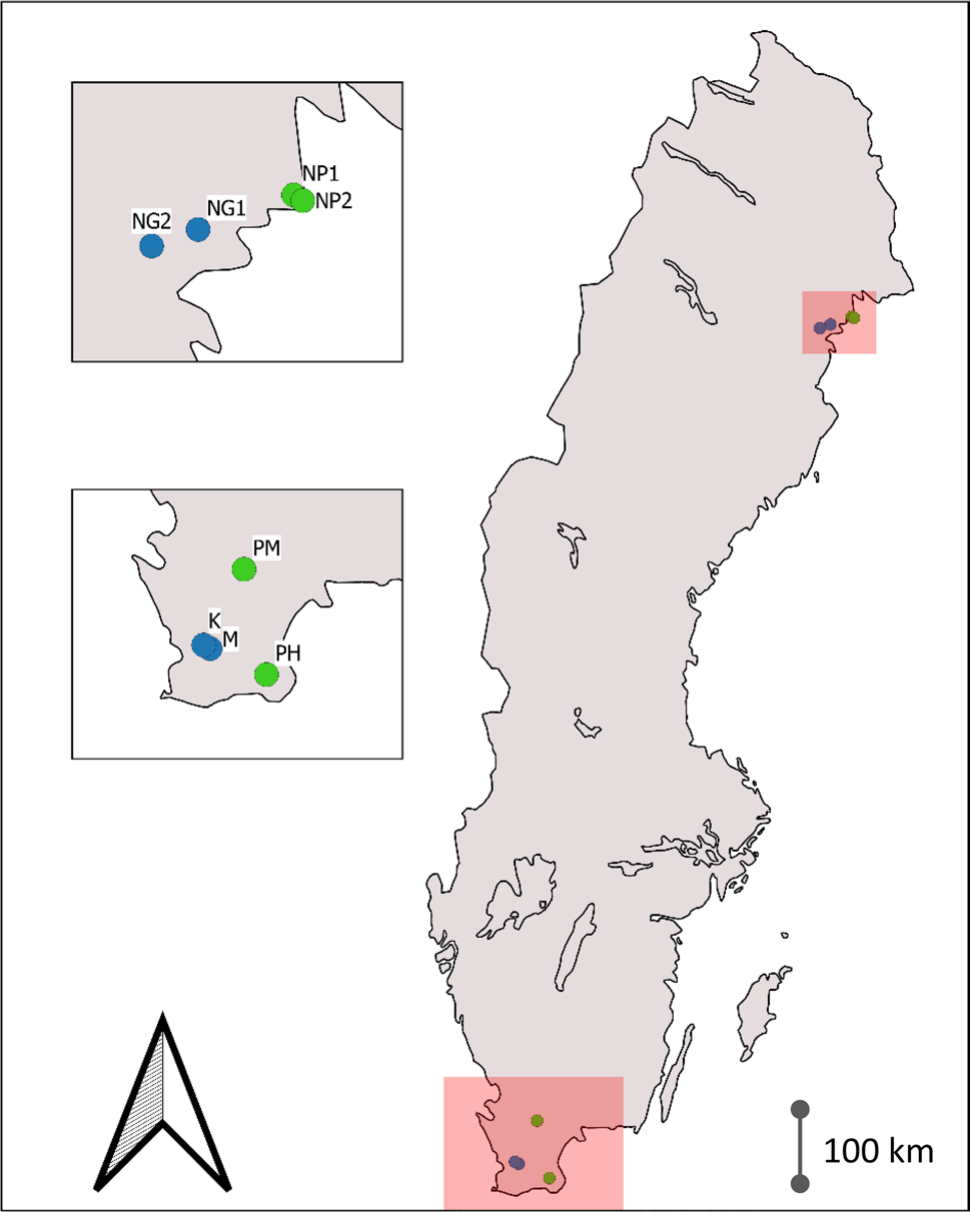
Map of Sweden showing the egg collection sites. Blue and green circles show *R. arvalis* and *B. bufo* sites, respectively

Eggs of both species were collected in April 2016 at two sites in Skåne county in southernmost Sweden and May 2016 at two sites in in Norrbotten county in northern Sweden (Fig. 1; Table S1). We collected ca. ten eggs from each of ten different clutches at each site. All collection ponds in the south were screened for Bd in 2015 and found negative (over 100 breeding adults tested; Meurling et al. 2020). In the north we tested in total four ponds in 2016 (two of which were included in the present study) and did not find Bd in any of them (Meurling et al. 2020).

The eggs and tadpoles were reared until metamorphosis in walk-in climate-controlled rooms at Uppsala University in plastic tanks filled with $20$ l reconstituted soft water (RSW; NaHCO_3_, CaSO_4_, MgSO_4_ and KCl added to deionized water; APHA 1985). Each clutch was kept in a separate tank under 18:6 h light/dark regime at 19 °C. The tadpoles were fed ad libitum spinach and fish flakes and water was changed every third day. At metamorphic climax (stage 42; Gosner 1960), the animals were moved to another tank of the same size with access to aquatic and terrestrial (aquarium sand) habitat and a shelter. Four days after completion of tail absorption (stage 46), the animals were transported to the sealed experimental facilities at the Swedish Institute for Veterinary Science, Uppsala, where they were kept individually in 1.2 l plastic tanks lined with moist paper towels and a lid of a plastic bottle as a shelter. The metamorphs were kept in these tanks until the end of the experiment and fed fruit flies and crickets ad libitum under 18:6 h light/dark regime at 19 °C. The condition of each animal was checked daily and the tanks were cleaned every third day.

### Infection experiment

The infection treatments were conducted after one week of acclimatization at the experimental facility. The experimental animals were exposed to one of two isolates of *Bd*-GPL (UK or SWE) or a sham infection consisting of culture medium (Table 1). The UK isolate (UKMal 01) was isolated from a wild alpine newt (*Icthyosaura alpestris*) in the UK in 2008. The Swedish isolate (SWED-40-5) originated from a wild green toad (*Bufotes viridis*) in Malmö municipality in southern Sweden in 2015. The animals were exposed individually for 5 h to 200 µl culture media containing a dosage of 60 000 zoospores in $30$ ml of RSW. The control group (C) was exposed for 5 h to an equivalent volume of RSW and culture media without *Bd* spores. Altogether, we treated 74 (25 in SWE, 24 in UK and 25 in C treatment) southern and 46 (16 SWE, 14 UK, 16 C) northern *Ra*. The corresponding numbers for *Bb* were 64 (21, 19, 24) southern and 90 (31, 31, 28) northern individuals.

**Table 1:** Number of experimental and infected individuals (as determined by qPCR) in different infection treatments for *R. arvalis* ($n=120$) and *B. bufo* ($n=154$)

Species	Region	Treatment	Total	Positive	%	Negative	%	Failed	%
***Rana arvalis***	North	Control	16	1	6.3	6	37.5	9	56.3
		*Bd*SWE	16	15	93.8	0	0.0	1	6.3
		*Bd*UK	14	13	92.9	0	0.0	1	7.1
	South	Control	25	2	8.0	18	72.0	5	20.0
		*Bd*SWE	25	24	96.0	0	0.0	1	4.0
		*Bd*UK	24	22	91.7	1	4.2	1	4.2
***Bufo bufo***	North	Control	28	1	3.6	16	57.1	11	39.3
		*Bd*SWE	31	28	90.3	0	0.0	3	9.7
		*Bd*UK	31	31	100.0	0	0.0	0	0.0
	South	Control	24	9	37.5	13	54.2	2	8.3
		*Bd*SWE	21	18	85.7	0	0.0	3	14.3
		*Bd*UK	19	14	73.7	1	5.3	4	21.1

After exposure the animals were monitored for 30 days. Animals showing irreversible signs of chytridiomycosis (loss of righting function) were euthanized with an overdose of MS222. Wet body mass was measured immediately before exposure and at the end of the experiment (or at death) with a microbalance to the precision of 0.1 mg. At the end of the experiment, the surviving animals were euthanized and stored in 96% ethanol at 4 °C.

### DNA extraction and qPCR analyses

To confirm infection status, we assessed the presence of *Bd* by using qPCR. DNA was extracted from a hind leg using a Prepman Ultra method described in Boyle et al. (2004). Presence of *Bd* was assessed by amplifying the internal transcribed spacer (ITS)-5.8S rRNA region (Boyle et al 2004). 25 µl reactions containing 12.5 µl 2 × Taqman Master Mix (Applied Biosystem, ref. 4,318,157), 2.25 µl 10 µM each of forward and reverse primers, 0.625 µl 10 µM MGB probe and 5µl of DNA (diluted × 10 in water) were run. Each sample was run in triplicate. An exogenous internal positive control (IPC; Hyatt et al. 2007) was added to one well in each triplicate (1 µl 10XExo IPC master mix and 0.5 µl 50XExo IPC DNA to each sample; VICTIM dye, Applied Biosystems ref. 4,304,662) to avoid false negatives due to inhibitors. The qPCR assays were run on a Biorad CFX96 Real Time System machine using amplification conditions described in Boyle et al. (2004) with standards of 0.1, 1, 10 and 100 genomic equivalents (GE). An individual was recorded as positive if at least one of the triplicate samples exhibited a positive signal (i.e. an exponential amplification curve). If the IPC showed signs of inhibition (i.e. was negative), the sample was rerun once. If the IPC was still negative the sample was assigned as failed and removed from the data set (Table 1). The above-mentioned standards were used to create a standard curve which was then used to calculate the infection intensity for each individual expressed in genome equivalents (GE).

For the statistical analysis of the infection, we used the log_10_ (zero values were replaced by 0.001, one tenth of the lowest measured non-zero value) of GE as a measure of infection load (IL). Molecular analyses of IL in 16 *Ra* and 23 *Bb* individuals failed (Table 1). Among the successful analyses, we found no presence of *Bd* for one *Ra* and one *Bb*, both of which were infected by the UK strain. All of these individuals were excluded from the statistical analyses involving IL.

### Statistical analyses

All analyses were conducted in R 3.5.2 (R Core Team 2018). Survival was analysed using Generalised Linear Models with a binomial error distribution and a logit link function. IL and growth were analysed using linear models. The assumptions for the linear models were checked using the model diagnostic plots in R, and these plots were also used to identify outliers. In one of the analyses, one outlier was removed (Table S1). In *Bb,* the growth data were log-transformed due to heteroscedasticity. Daily growth was defined as (mass at day 30—mass at exposure)/30 in both species. In order to be able to study the non-lethal effects of *Bd* infection, dead individuals (3 *Ra* and 55 *Bb*) were removed from the growth analyses.

The models that best explained differences in IL, survival and growth were selected using 170 bidirectional eliminations (*stepAIC* function in R package *MASS*) starting from the full model: Response ~ Region + Bd-infection + Size at infection + IL + Interactions, where region and infection were treated as factors and size and IL as covariates. The models were first run investigating the effect of Bd-infection by grouping the two *Bd* treatments together and comparing them with the control treatment (factor levels: infected and controls), and second by comparing the effects of the two Bd-strains (UK and SWE) excluding the controls from the model. If any interactions were included in the selected model, but none of them was close to significance (p < 0.1), the model selection was rerun without interactions to avoid unnecessarily complicated model. Type III sums of squares were used when interactions were included (*Anova* function in R package *car*). Covariates were standardized with Student’s method before the analyses.

## Results

The qPCR analyses showed high infection success: only two of the successfully analysed individuals from the exposed groups were negative to *Bd* infection at the end of the experiment (one *Bb* and one *Ra*; Table 1) and were removed from the analyses. Thirteen control individuals were also found *Bd* positive (Table 1). However, IL in these individuals was always very low (mean 0.08 ± 0.014 (SE) genomic equivalents, range 0.03–0.235), and we find it likely that these samples were contaminated during sample processing at the end of the experiment. For the statistical analysis, IL in these individuals was therefore considered to be 0.

**Fig. 2:**
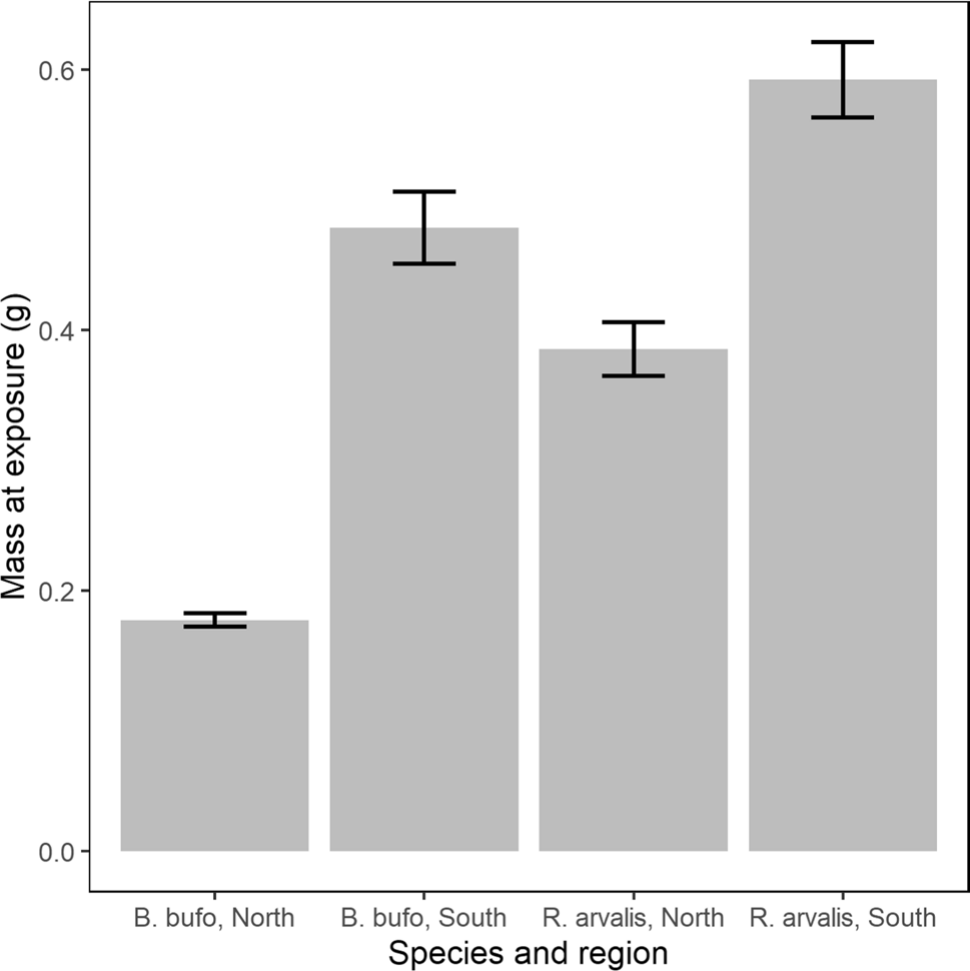
Mass at exposure (g ± SE) for *R. arvalis* and *B. bufo* at each region ($n=174$)

At the time of *Bd* infection, northern animals were significantly smaller than southern animals both in *Ra* (*F*_1, 118_ = 26.35, p < 0.001) and in *Bb* (*F*_1, 152_ = 159.14 p < 0.001; Fig. 2). There were no size differences between the infection treatments at the time of infection (*Ra*: *F*_2, 117_ = 0.09, p = 0.91, *Bb*: *F*_2, 151_ = 0.09, p = 0.916).

### Infection load

**Fig. 3:**
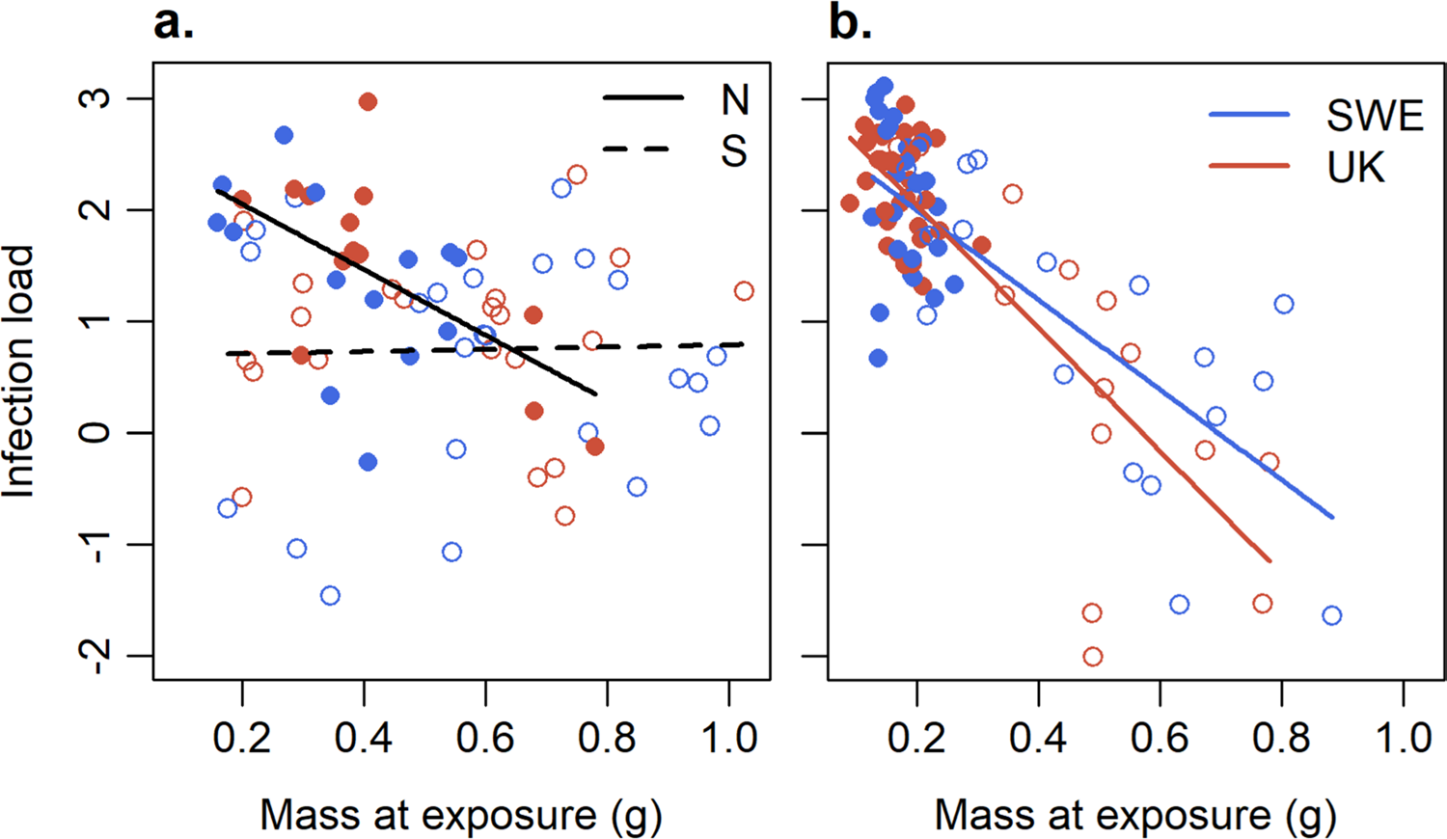
Infection load (log10 average GE, at the end of treatment) as the function of size at infection with SWE or UK *Bd* strain in **a*** R. arvalis* ($n=73$) and **b** *B. bufo* ($n=91$). Lines give the predictions of the model. Filled dots and solid lines represent the northern region (N), while open dots and dashed lines represent the southern region (S)

For *Ra,* the selected model was IL ~ Size + Region + Size × Region (Table S1a). We found a significant interaction between size at infection and region (*F*_1, 69_ = 6.5, p = 0.013), size having a negative effect on IL in the northern region, but no effect in the southern region (Fig. 3a).

*Bb* had higher IL than *Ra* (*F*_1, 163_ = 15.09, p < 0.001; Fig S1). The selected model for *Bb* was IL ~ *Bd*-strain + Size + *Bd*-strain × Size (Table S1b). Size had a significant negative effect on infection load (*F*_1, 87_ = 64.45, p < 0.001, Fig. 3b). The interaction between *Bd*-strain and size was close to significant (*F*_1, 87_ = 3.66, p = 0.059), large toadlets infected with SWE strain having somewhat higher loads than large individuals infected with UK strain.

### Survival

**Fig. 4:**
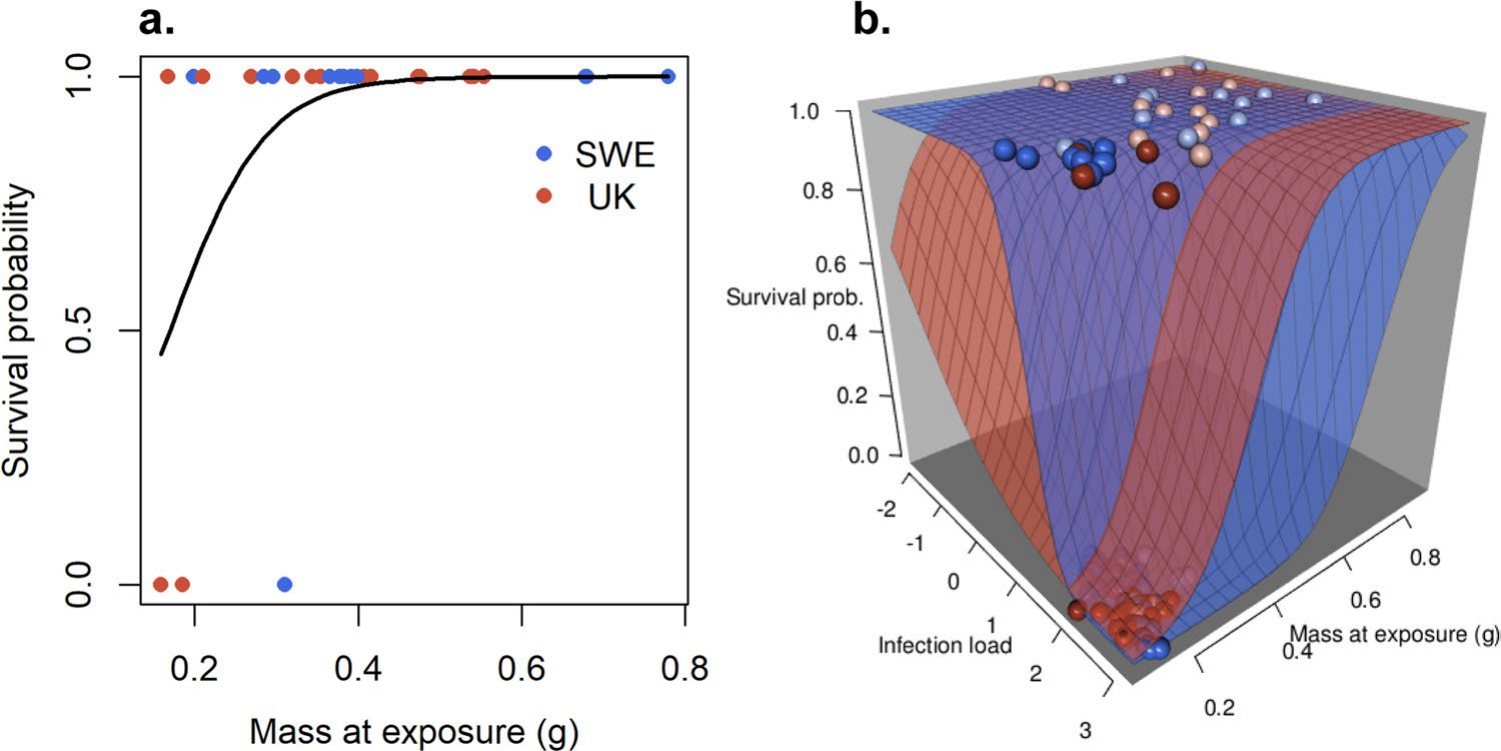
Survival as a function of size at infection with SWE or UK *Bd* strain for **a** *R. arvalis* from the northern region ($n=28$). **b** Survival in *B. bufo* as function of infection load and size at infection, where dark and pale dots represent the northern and southern regions, respectively ($n=91$). Curve (**a**) and surfaces (**b**) give the predictions of the model. Blue dots and surface (in **b**) refer to SWE strain and red dots and surface to UK strain. In **b** some pale dots are hidden among the dark dots in the lower corner in the front (high infection load and small size)

Survival in southern *Ra* was complete, whereas three infected individuals from the northern region died during the experiment, resulting in 92.9% survival in UK and 87.5% in SWE treatment. As survival was complete in the control treatment and in the southern region these were excluded from the statistical analyses. The selected model for the infected individuals in the northern region was Survival ~ Size (Table S3a) indicating poorer survival of smaller individuals in the two infection treatments in the northern region (χ^2^_1, 26_ = 6.49 p = 0.011; Fig. 4a). However, this result should be interpreted with caution as it is based on only three cases of mortality.

While all *Bb* in the control treatment survived the experiment, there was considerable mortality in the infection treatments. Furthermore, survival was higher in the southern (66.7% in SWE and 89.5% in UK) than in the northern region (38.7% in SWE and 12.9% in UK). Due to 100% survival in the control treatment we excluded it from the full model. We also excluded the interaction term between region and size, as well as between region and IL, as the size and IL range in the northern region covered only a small subset of the range in the southern region, and including these interactions may have led to problematic extrapolations. Survival of *Bb* in the two infection treatments was best explained by the model Survival ~ Bd-strain + Size + IL + *Bd*-strain × IL (Table S3b). Initial size had a strong positive effect on survival (*F*_1, 86_ = 13.10, p < 0.001, Fig. 4b), whereas IL had a strong negative effect (*F*_1, 86_ = 27.61, p < 0.001). In addition, the significant interaction between *Bd* strain and infection load (*F*_1, 86_ = 7.05, p = 0.009) was due to SWE strain causing higher mortality at high IL than UK strain (Fig. 4b).

### Growth

**Fig. 5:**
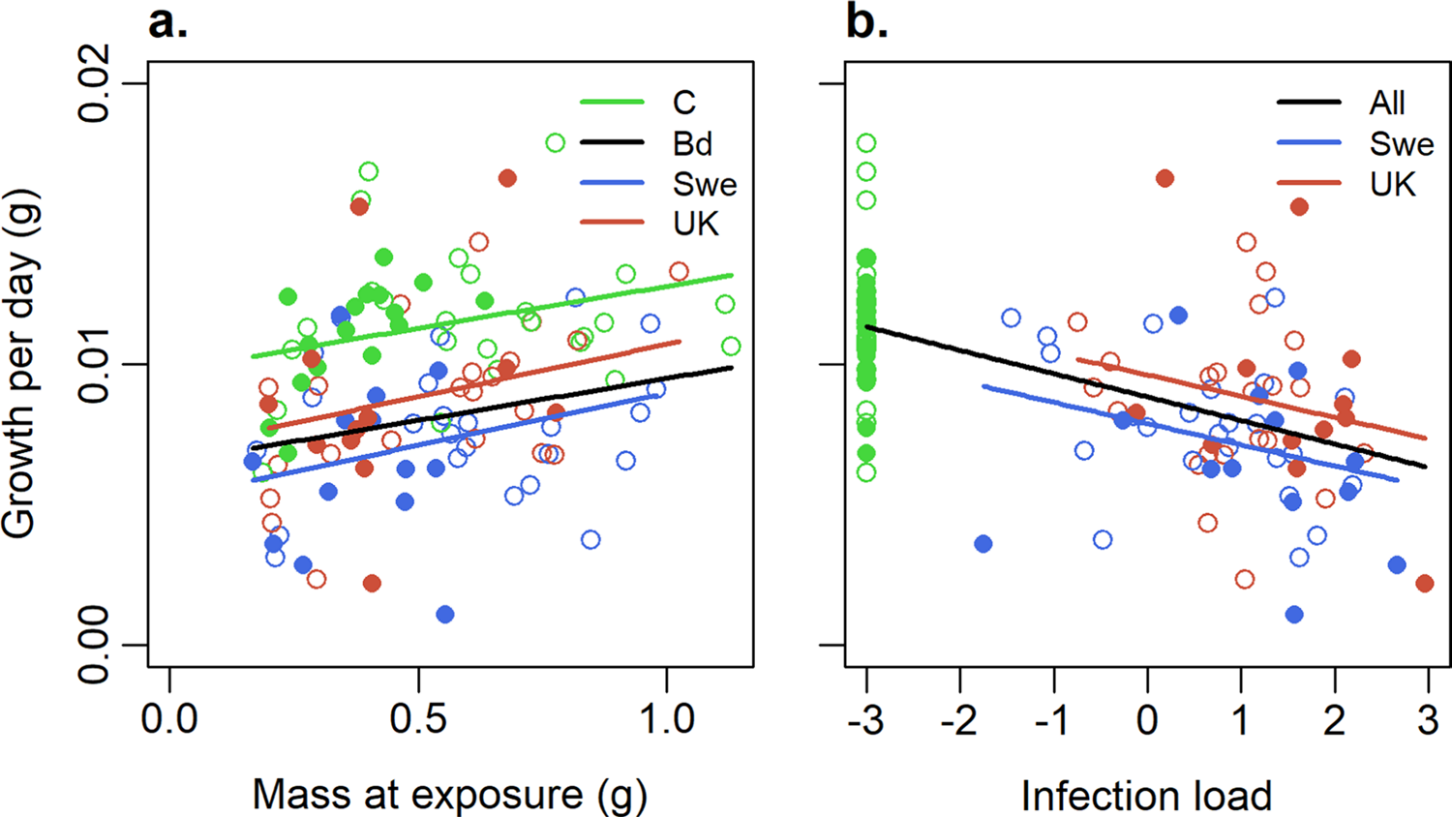
Growth (g/day) of *R. arvalis* as a function of **a** size at infection in different infection treatments, and **b** infection load (log10 average GE), at the end of experiment. Filled and open dots, and solid and dashed lines, represent the northern and southern regions, respectively. The blue and red lines give the predicted growth of individuals infected with SWE and UK strains, respectively (model in Table S4b, $n=71$), evaluated for average infection load in **a** and average size in **b**. The black and green line gives the predicted growth, evaluated for average infection load of the infected and control treatments, respectively, in **a**, while the black line is evaluated average size of all individuals in **b** (model in Table S4a, $n=112$)

The model explaining growth best for *Ra* was Growth ~ IL + Size (Table S4a) showing that larger individuals had higher growth rates (*F*_1,109_ = 7.76, p = 0.006; Fig. 5a), and individuals with higher infection load suffered a more severe growth decline (F_1,109_ = 46.6, p < 0.001; Fig. 5b). An additional analysis showed that this was due to infected individuals (combining the two Bd-strains and removing IL from the model) having lower growth than those in the control treatment (*F*_1,113_ = 38.1, p < 0.001). The comparison between the two Bd-strains revealed that individuals infected with SWE strain have lower growth than those infected with UK strain (*F*_1,67_ = 6.95, p = 0.01; Fig. 5b, Table S4b) and that a higher infection load results in a more severe growth decline (*F*_1,67_ = 5.10, p = 0.027; Fig. 5b, Table S4b).

**Fig. 6:**
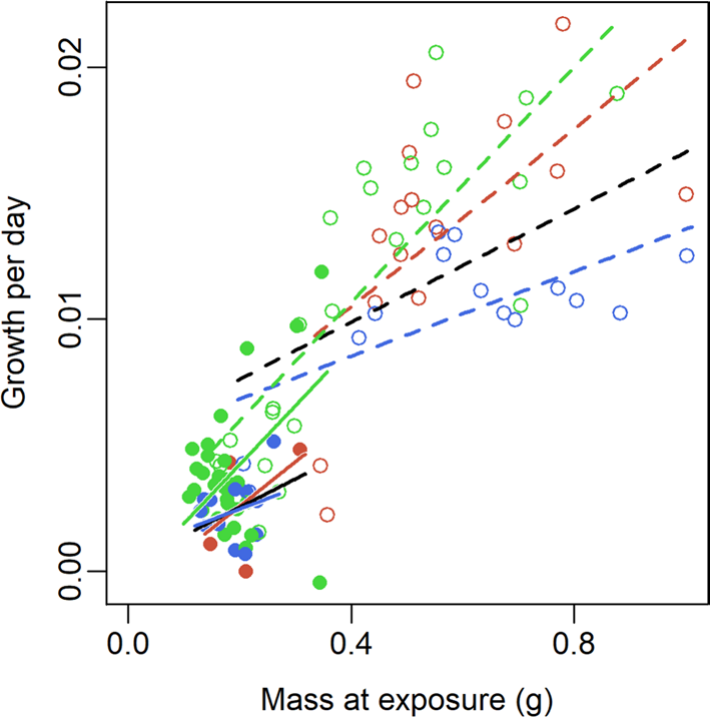
Growth (g/day) of *B. bufo* as a function of size at infection with SWE or UK *Bd* strain or in the control treatment. Filled and open dots, and solid and dashed lines, represent the northern and southern regions, respectively. The black and green lines give the predicted growth of infected and control treatments, respectively (model in Table S4c, $n=98$). The blue and red lines give the predicted growth of individuals infected with SWE and UK strains, respectively (model in Table S4d, $n=46$)

The model best explaining growth in *Bb* was Growth ~ Bd-infection + Region + Size + Bd-infection × Region + Bd-infection × Size (Table S4c). Larger *Bb* had higher growth rate (F_1,92_ = 56.15, p < 0.001; Fig. 6). Bd-infection reduced growth (F_1,92_ = 9.03, p = 0.003; Fig. 6) and the positive size effect was weaker in *Bd*-infected individuals (Infection × Size: F_1,92_ = 8.22, p = 0.005; Fig. 6). When analysed separately as a covariate, IL explained the growth decline independent of individual size and region (F_1,85_ = 5.65, p = 0.020, data not shown). The absence of interactions between infection load and both size and region is explained by IL indirectly considering both size and region, as IL was lower in larger individuals, and as northern individuals were smaller.

Including IL as a covariate in the model comparing *Bb* growth in the two infection treatments resulted in a large, possibly overfit model with four significant interactions but sample size of only 37. We therefore run the model selection without IL (Table S4d). We found that individuals infected with SWE were more negatively affected than those infected with UK and this effect was stronger in large individuals (Strain × Size: F_1,41_ = 6.15, p = 0.017; Fig. 6). Northern individuals had lower growth than those from the south (F_1,41_ = 9.87, p = 0.003; Fig. 6).

## Discussion

We found that *Bd* infection lowered survival especially in *Bb* and in the northern region. Our analyses suggest that the survival differences between the regions were largely mediated by body size, smaller individuals being more sensitive to *Bd*. While the results were qualitatively similar for *Ra*, these results should be interpreted with caution due to considerably lower mortality in this species. Furthermore, we found that *Bd* infection led to sub-lethal effects in terms of reduced growth, suggesting that individuals surviving the infection may have lower fitness mediated by their smaller body size. These results indicate that *Bd* infection may have both direct and indirect effects on amphibian populations and that high- latitude populations may run a higher risk of negative effects than their low-latitude counterparts.

Both species became infected in our experiment, but IL in *Ra* was lower and *Bd*-mediated mortality was only a fraction of the mortality experienced by *Bb*. However, *Ra* individuals did not clear the infection during the 30 day observation period. These results agree with previous studies showing that brown frogs have higher tolerance to *Bd*, while bufonids are more susceptible to *Bd*-infection (Bosch and Martínez-Solano 2006; Garner et al. 2009; Gahl et al. 2012; Balaz et al. 2014; Bielby et al. 2015). When comparing susceptibility to infection and *Bd*-mediated mortality between two anuran species, Bielby et al. (2015) found that *R. temporaria,* a sister species to *Ra*, was resistant to infection even at high doses, while almost all *Bb* became infected and showed high dose-dependent mortality. Since *Ra* has higher infection prevalence in the wild (Meurling et al. 2020) and higher infection tolerance (this study), we suggest that *Ra* may act as a reservoir species and a possible vector for *Bd*-transmission to more sensitive species such as *Bb*. Indeed, Kärvemo et al. (2019) showed that *Bb* populations coexisting with *Ra* had higher *Bd*-prevalence than populations breeding in ponds without *Ra*.

We found a clear difference in survival between northern and southern populations especially in *Bb*. Northern individuals were smaller at the time of infection than southern individuals, and our analyses suggest that the survival difference was mainly mediated by body size. As we raised the tadpoles under common garden conditions, the differences in body mass most likely have a genetic origin. The higher vulnerability of smaller individuals to *Bd* agrees with previous studies (Garner et al. 2009; Bradley et al. 2015; Burrow et al. 2017). Smaller individuals may have less resources and less developed immune system to fight the infection increasing their vulnerability to disease (Møller et al. 1998; Lochmiller and Deerenberg 2000; Burrow et al. 2017). This is supported by the fact that smaller individuals had higher ILs. Smaller individuals may also be more vulnerable to *Bd*-mediated water loss as they have larger surface area to body mass ratio. Increased water loss via sloughing is an important symptom in chytridiomycosis, which may render smaller individuals more sensitive to *Bd* infection (Russo et al. 2018; Wu et al. 2019).

In addition to the size differences, two further, not mutually exclusive, explanations may further explain higher mortality in the northern populations. Firstly, northern populations may have less effective immune systems because of reduced genetic variation due to postglacial colonization processes (Hewitt 2000, see Cortazar-Chinarro et al. 2017, Rödin-Mörch et al. 2019 for *Ra*, Thörn et al. 2021 for *Bb*) or lower pathogen abundance at higher latitudes (Schemske et al. 2009). This hypothesis gains support from the fact that MHC variation in both our study species is lower at higher latitudes (Cortázar-Chinarro et al. 2017, 2022). Moreover, *Bd*-mediated survival in *Bb* seems to be linked with certain MHC alleles (Cortázar-Chinarro et al. 2022), as also found in other species (Savage and Zamudio 2011; Savage et al. 2018; Kosch et al. 2019). Secondly, higher larval development rates in the northern populations may trade off with disease resistance (Johnson et al. 2011; Woodhams et al. 2016). Also this hypothesis is indirectly supported by the facts that more time-constrained populations have higher development rates in both our study species (Luquet et al. 2015, 2019) and that *Ra* tadpoles experimentally induced to develop faster have weaker immune response (Murillo-Rincon et al. 2017). Additional studies focusing on *Bd* resistance in known MHC and developmental genotypes would be highly interesting.

*Bd* infection had clear negative effects on growth in both species. As body size is positively related to fitness in juvenile amphibians (Earl and Whiteman 2015), these results suggest that *Bd* may have sublethal fitness effects. For example, hibernation success is often positively related to body size and failing to reach a sufficient size before hibernation can greatly reduce overwinter survival (Altwegg and Reyer 2003). This can be especially detrimental at higher latitudes where the hibernation period can reach eight months. Small body size may also lead to higher risk of predation, delayed maturation and lower ability to compete for resources and mates (reviewed in Earl and Whiteman 2015). In the long run, these effects may decrease population growth rate and ability to cope with environmental changes such as higher temperature due to climate change. In our case, even if survival of *Ra* was not strongly affected by *Bd* infection, the sublethal effects of infection mediated by body size may still lower individual and population fitness.

We found relatively little evidence for differences in pathogenicity between the two *Bd* isolates. Nevertheless, we found significant strain × size interaction in survival of *Bb* with larger toadlets being more vulnerable to infection with SWE strain than with UK strain. These results suggest that individuals infected with UK strain may relatively quickly reach a size where the lethality of *Bd* is reduced, while *Bd*-mediated mortality induced by SWE strain is less size-dependent. This effect can be especially clear in the southern individuals which are larger at metamorphosis, while the smaller northern individuals stay longer in the vulnerable size classes. Importantly, in both host species SWE strain had a stronger negative effect on growth, suggesting that SWE infected individuals may spend a longer time in the vulnerable size class, and that potential sublethal fitness effects mediated by body-size differences are more severe in SWE infection. The reasons for the virulence differences between the BdGPL strains are unclear, but earlier studies have emphasized the importance of zoospore production and sporangia size as well as differences in genes related to colonization ability and cell invasiveness (Fisher et al. 2009b; Greener et al. 2020). Moreover, the host species from which the *Bd* strain was isolated can play a role (Belasen et al. 2022). Interestingly, in a recent meta-analyses Belasen et al. (2022) found that the geographical proximity of the BdGPL source locality and the host population played very little role in *Bd*-induced mortality.

A potential caveat in our study is that we used laboratory-raised (but wild-collected) individuals. Lab-raised individuals may not have as diverse skin microbiota as individuals living in the wild which are exposed to more diverse microbial community. Indeed, captive amphibians often have a reduced and less varied bacterial community than wild populations of the same species (Antwis et al. 2014; Bataille et al. 2016). As skin microbiome plays an important role in defending against fungal and other pathogens, this could impact the ability of amphibians reared in captivity to respond to *Bd* infection (Harris et al. 2009; Walke et al. 2015; Madison et al. 2017; Woodhams et al. 2018). We currently lack knowledge on the skin microbiomes of our study species and if these differ between geographical regions. Microbiome studies are needed for additional insight on factors behind the high mortality found in this study.

*Bd* is widespread in the southern parts of Sweden (Kärvemo et al. 2018, 2019; Meurling et al. 2020). However, in a pattern similar to much of Europe (Lips 2016; Scheele et al. 2019), no cases of chytridiomycosis or unusual die-offs have been found in Sweden. Our experimental results suggest that even though no negative effects of the infection have been seen in the wild, this might not be the complete picture. It is currently unclear how well the present results translate to natural conditions, but we note that *Bd* causes sublethal effects in terms of reduced movements and body condition in wild Scandinavian amphibians (Kärvemo et al. 2019, 2020). Furthermore, the lethality of *Bd* is highly dependent on environmental conditions, including temperature (e.g., Novakowski et al. 2016, Mosher et al. 2018, Cohen et al. 2019), and relatively minor elevations in mortality may risk long-term survival of *Bd*-infected amphibian populations (Muths et al. 2011; Spitzen-van der Sluijs et al. 2017). Two important conclusions can be drawn. Firstly, very few *Bd* surveys have been conducted in northernmost Scandinavia, and the results so far have been negative (Meurling et al. 2020). As populations at higher latitudes can be more vulnerable to infection, it is important to investigate the occurrence of *Bd* in these areas and, if still possible, prevent or limit the possible northward spread of the fungus. Secondly, we showed that infection leads to higher mortality and reduced body size. These, in turn, can lead to reduced population growth rates in the long-term even in the absence of major mortality effects. As the potential negative effects of *Bd* on population growth can be relatively subtle and difficult to detect (Doddington et al. 2013, Spitzen-van der Sluijs et al. 2017, Mosher et al. 2018), long-term monitoring of amphibian populations is of high importance.

### Supplementary Information

Below is the link to the electronic supplementary material.

Supplementary file1 (DOCX 90 KB)

## Data Availability

The data will be deposited in DRYAD upon acceptance.
