# Supplementary material for: Body size mediates latitudinal population differences in the response to chytrid fungus infection in two amphibians
Source: Oecologia. 2023 Dec 14;204(1):71–81. doi: 10.1007/s00442-023-05489-5 (PMC10830819; doi:10.1007/s00442-023-05489-5)
Supplement: Supplementary file 1 — Supplementary file1 (DOCX 90 KB) [file 442_2023_5489_MOESM1_ESM.docx]

Supplementary material to:

**Body size mediates latitudinal population differences in the response to chytrid fungus infection in two amphibians**

Sara Meurling^1^, Mattias Siljestam^1^_,_ Maria Cortazar-Chinarro^1,2,3^, David Åhlen^4^, Patrik Rödin-Mörch^1^, Erik Ågren^5^, Jacob Höglund^1^ and Anssi Laurila^1^*

^1^Animal Ecology/ Department of Ecology and Genetics, Uppsala University, Sweden

^2^MEMEG/Department of Biology, Lund University, Lund, Sweden

^3^Department of Earth, Ocean and Atmospheric Sciences, University of British Columbia, Vancouver, Canada

^4^De[partment of Ecology, Environment and Plant Sciences](http://www.su.se/deep/english), Stockholm University, Sweden

^5^Department of Pathology and Wildlife Diseases, National Veterinary Institute, Uppsala, Sweden

*Corresponding author: anssi.laurila@ebc.uu.se

**Authors’ contributions:** SM, MCC, JH and AL conceived and designed the experiments, SM, MCC and DÅ performed the experiments and EÅ provided advice and logistic help, SM, PRM and MS analysed the data, SM and AL wrote the paper with input from all the authors.

**Table S1**. Coordinates for the collection sites

| Species | Region | Population | N | E |
| --- | --- | --- | --- | --- |
| *Rana arvalis* | North | NG1 | 65.519974 | 21.685978 |
|  |  | NG2 | 65.488998 | 21.378151 |
|  | South | M | 55.699774 | 13.360416 |
|  |  | K | 55.722114 | 13.284693 |
| *Bufo bufo* | North | NP1 | 65.583139 | 22.319458 |
|  |  | NP2 | 65.56554 | 22.37404 |
|  | South | PM | 56.217897 | 13.731548 |
|  |  | PH | 55.539031 | 14.009702 |

Table S2. Results from final general linear models on infection load. a) *R. arvalis* ($n=73$, one outlier removed)*,* b) *B. bufo* ($n=91$)

|  | Sum of squares | Df | *F* | *P* |
| --- | --- | --- | --- | --- |
| Size | 5.5 | 1 | 7.54 | **0.008** |
| Region | 8.8 | 1 | 12.11 | **< 0.001** |
| Size x Region | 4.7 | 1 | 6.50 | **0.013** |
| Residuals | 50.3 | 69 |  |  |

|  | Sum of squares | Df | *F* | *P* |
| --- | --- | --- | --- | --- |
| Bd-strain | 0.8 | 1 | 1.70 | 0.196 |
| Size | 31.9 | 1 | 64.45 | **< 0.001** |
| Strain x Size | 1.8 | 1 | 3.66 | 0.059 |
| Residuals | 43.0 | 87 |  |  |

Table S3. Results from final generalized linear models on survival. a) *R. arvalis*: the analyses only cover the northern population and the two *Bd*-treatments as survival in the southern population and control treatment were complete ($n=28$). b) *B. bufo:*  only the two *Bd*-treatments are included as survival was complete in the control treatment ($n=91$).

|  | LR chisquared | Df | *P* |
| --- | --- | --- | --- |
| Size | 6.5 | 1 | **0.011** |
| Residuals | 12.6 | 26 |  |

|  | Sum of squares | Df | *F* | *P* |
| --- | --- | --- | --- | --- |
| Bd-strain | 0.2 | 1 | 0.24 | 0.624 |
| Size | 9.3 | 1 | 13.10 | **< 0.001** |
| Infection load (IL) | 19.7 | 1 | 27.61 | **< 0.001** |
| Strain x IL | 5.0 | 1 | 7.05 | **0.009** |
| Error | 61.3 | 86 |  |  |

Table S4. Results from general linear models on growth. a*) R. arvalis* all alive individuals ($n=112$, excluding the three dead individuals), b) *R. arvalis*, alive *Bd*-infected individuals ($n=71$, excluding the three dead individuals), c) *B. bufo* all alive individuals ($n=98$, excluding 55 dead individuals), d) *B. bufo*, *Bd*-infected individuals only ($n=46$, excluding 55 dead individuals),

|  | Sum of squares | Df | *F* | *P* |
| --- | --- | --- | --- | --- |
| Infection load (IL) | 0.000328 | 1 | 46.59 | < **0.001** |
| Size | 0.000055 | 1 | 7.76 | **0.006** |
| Residuals | 0.000766 | 109 |  |  |

*b)*

|  | Sum of squares | Df | *F* | *P* |
| --- | --- | --- | --- | --- |
| Infection load (IL) | 0.000038 | 1 | 5.10 | **0.027** |
| *Bd*-strain | 0.000052 | 1 | 6.95 | **0.010** |
| Size | 0.000049 | 1 | 6.61 | **0.012** |
| Residuals | 0.000501 | 67 |  |  |

*c)*

|  | Sum of squares | Df | *F* | *P* |
| --- | --- | --- | --- | --- |
| *Bd*-infection | 0.000088 | 1 | 0.03 | **0.003** |
| Region | 0.000022 | 1 | 2.32 | 0.131 |
| Size | 0.000545 | 1 | 56.15 | **< 0.001** |
| Bd-infection x Region | 0.000031 | 1 | 3.20 | 0.077 |
| Bd-infection x Size | 0.000080 | 1 | 8.22 | **0.005** |
| Residuals | 0.000892 | 92 |  |  |

*d)*

|  | Sum of squares | Df | *F* | *P* |
| --- | --- | --- | --- | --- |
| *Bd*-strain | 0.000024 | 1 | 2.90 | 0.096 |
| Region | 0.000081 | 1 | 9.87 | **0.003** |
| Size | 0.000067 | 1 | 8.17 | **0.007** |
| Bd-strain x Size | 0.000050 | 1 | 6.15 | **0.017** |
| Residuals | 0.000335 | 41 |  |  |


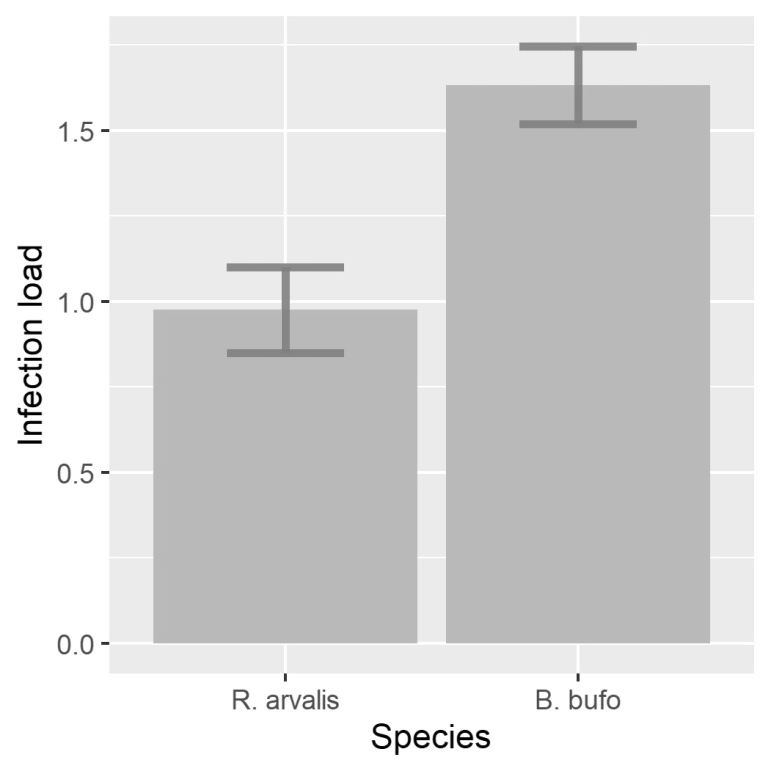


**Fig. S1.** Infection load (genomic equivalents) in *R. arvalis* ($n=115$) and *B. bufo* ($n=143$)
